# Commentary: Comprehensive lipidome profiling of the kidney in early-stage diabetic nephropathy

**DOI:** 10.3389/fendo.2022.1015305

**Published:** 2022-09-13

**Authors:** Katsumi Iizuka

**Affiliations:** Department of Clinical Nutrition, Fujita Health University, Toyoake, Japan

**Keywords:** kidney, diabetic kidney disease, C18:2, C18:3, lipidome, C16:0, C18:1

## Introduction

Early detection of kidney disease is very important, and in clinical practice, urinary albumin is measured in diabetic patients. However, many conditions that worsen renal function (eGFR) without increasing urinary protein are observed, giving the impression that diabetic kidney disease is a mixture of multiple conditions (1). Therefore, multiple biomarkers are needed to determine the mixture of multiple pathologies. Moreover, early intervention for diabetic kidney disease requires prevention of end-stage renal failure. For this reason, a biomarker to identify early-stage diabetic kidney disease is needed (2).

In the kidney, the accumulation of excess fatty acids results in damage to podocytes, proximal tubules, and tubulointerstitial tissue by reactive oxygen species and lipid peroxides, causing mitochondrial dysfunction, inflammation, and injury to the glomeruli and tubulointerstitial tissue (3, 4). In addition to excessive blood uptake in hyperlipidemia, dysregulation of fatty acid synthesis and inhibition of β-oxidation, probably by SREBP1c and ChREBP, is also a pathogenesis of fatty acid accumulation (5, 6). Thus, excess accumulation of lipids in the kidney has important implications in renal disease. Phospholipids also play a role in regulating cellular membrane stability and cell signaling. Shingolipid accumulation was also reported to contribute to the development of diabetic kidney disease. For this reason, blood phospholipids and sphingomyelin have been studied in patients with kidney disease, but no studies have been conducted on early-stage kidney disease.

## Characteristic lipid profiles in the kidney cortex of diabetic kidney disease

### Increased lipid contents in the kidney cortex of diabetic rats

In 2020, Biyu Hou et al. published a new lipidome paper in Frontiers in Endocrinology (Research Topic: Advances in the Research of Diabetic Nephropathy) (7). The present report by Biyu Hou et al. was performed on STZ-treated rats fed a high-fat, high-sucrose diet. This model showed early-stage diabetic kidney disease with microalbuminemia, decreased creatinine clearance, basement membrane chicking, and glomelular hypertrophy (7). Moreover, hypertriglyceridemia and hypercholesterolemia were observed. A comprehensive analysis of multiple lipids in the renal cortex was performed, examining 437 lipids. In this study, first, there was an increase in free fatty acids, triacylglycerol, diacylglycerol, cholesterol, and cholesteryl esters, a decrease in phosphatidylethanolamine and phosphatidylserine, and significant increases in lysophospholipids and sphingolipids. Thus, high fat diet feeding caused to increase C16:0 and C18:1 level in FFAs, diacylglycerol, triacylglycerol, cholesteryl esters. Furthermore, the increases in C16:0 and C18:1 may be associated with increased conversion of C16:0 to C18:1 by increased EVOLV6 and SCD-1 activity through activation of SREBP1c and ChREBP (5, 8). Although the general results are consistent with those reported previously, there are many interesting aspects from a nutritional point of view.

### C18:2 may be a biomarker in early-stage diabetic kidney disease

Of note is the increase in C18:2 in the side chains of diacylglycerols and cholesterol esters as well as free fatty acids; C18:2 is an essential fatty acid found in vegetable oils and is used in the synthesis of arachidonic acid. In fact, since C18:2 cannot be synthesized *in vivo*, overexpression of transcription factors such as ChREBP does not alter C18:2 levels (8). In this paper, and in plasma lipidome analysis in a human study, increases in blood C18:2 are seen with both C16:0 and other fatty acids in patients with diabetic nephropathy (7). Moreover, a recent study showed that tetranor-prostaglandin E metabolite, an arachidonic acid metabolite, was significantly higher in subjects with early stage nephropathy than in healthy subjects and increased with the progression of nephropathy (9). In contrast, it has also been reported that people who consume low amounts of fish oil and safflower oil are at higher risk for chronic kidney disease (10). C18:2 is a fatty acid of dietary origin and a source of arachidonic acid, which is closely associated with inflammation. C18:2 is the source of arachidonic acid, which is closely associated with inflammation. In contrast, C18:3 is converted into eicosapentaenoic acid (C20:5) and docosahexaenoic acid (C22:6). C18:3 also increased; however, the absolute increase was also less than that of C18:2. In a cross-sectional study of dietary n-3 and n-6 fatty acids and inflammatory markers, there were statistically significant inverse associations between n-3 fatty acid intake and inflammatory markers (10). At low levels of n-3 fatty acid intake, n-6 fatty acids are associated with high levels of inflammatory markers (11). This may reflect the fact that this is due to the composition of the diet used in the experiment and study. Therefore, it should be noted that if experiments were conducted using different diet compositions, different lipidome results may also be observed. In fact, it has been reported that consumption of a fish diet high in n-3 fatty acids lowers the C18:2 content of cardiolipin in WAT and BAT compared to a high lard diet (12). Thus, there is a need to clarify this point, including the fact that C18:2 is a fatty acid of dietary origin, the amount consumed, and the ratio of C18:2 to C18:3.

Another concern is that this is an STZ-treated model, and other diabetic nephropathy models need to be tested to see if there is indeed no effect of STZ itself on renal damage. It seems necessary to examine what changes occur in the kidneys of the high-fat, high-sucrose diet model without STZ. It also seems that there is room for further study on how nephropathy can be affected by feeding a diet with reduced C18:2.

## Perspective

This study comprehensively analyzed phospholipids, lyso-phospholipids, and sphingolipids in addition to free fatty acids, diacylglycerol, triacyglycerol, and cholesteryl esters in diabetic kidneys, which may lead to the elucidation of the pathogenesis of diabetic kidney disease and the discovery of biomarkers in the future. In addition, once we know the functional aspects of each lipid molecular species (e.g. PC 16:0/18:2) rather than lipid species (e.g. PC 34:2), we will be able to further understand the meaning of these data. Finally, when interpreting the results, one must bear in mind that the quality and quantity of lipids in the diet will affect the tissue content of lipids.

## Author contributions

The author confirms being the sole contributor of this work and has approved it for publication.

## Funding

This work was supported by grants from Japan Society for the Promotion of Sciences (JSPS) [17K00850] and The Public Foundation of Elizabeth Arnold-Fuji.

## Conflict of interest

The author declares that the research was conducted in the absence of any commercial or financial relationships that could be construed as a potential conflict of interest.

## Publisher’s note

All claims expressed in this article are solely those of the authors and do not necessarily represent those of their affiliated organizations, or those of the publisher, the editors and the reviewers. Any product that may be evaluated in this article, or claim that may be made by its manufacturer, is not guaranteed or endorsed by the publisher.
